# Preprocedural circulating galectin-3 and the risk of mortality after transcatheter aortic valve replacement: a systematic review and meta-analysis

**DOI:** 10.1042/BSR20202306

**Published:** 2020-09-16

**Authors:** Hong-liang Zhang, Guang-yuan Song, Jie Zhao, Yu-bin Wang, Mo-yang Wang, Yan-lu Xu, Bin-cheng Wang, Guan-nan Niu, Zhi-hong Liu, Yong-jian Wu

**Affiliations:** Fuwai Hospital, National Center for Cardiovascular Diseases, Chinese Academy of Medical Sciences and Peking Union Medical College, Beijing, China; Beilishi Road 167, Xicheng District, Beijing 100037, China

**Keywords:** Aortic stenosis, Galectin-3, Meta-analysis, Mortality, Transcatheter aortic valve replacement

## Abstract

**Background:** Galectin-3 may predict mortality for patients with aortic stenosis (AS) after transcatheter aortic valve replacement (TAVR). However, the results were inconsistent. We aimed to evaluate the association between baseline galectin and mortality after TAVR in a meta-analysis.

**Methods:** Related follow-up studies were obtained by systematic search of PubMed, Cochrane’s Library, and Embase databases. Both the fixed- and the random-effect models were used for the meta-analysis. Subgroup analyses were performed to evaluate the influences of study characteristics on the outcome.

**Results:** Five prospective cohort studies with 854 patients were included, with a follow-up period between 1 and 1.9 years. Patients with higher baseline circulating galectin-3 had an increased risk of all-cause mortality after TAVR (random-effects model: risk ratio [RR]: 1.63, 95% confidence interval [CI]: 1.19–2.23, *P*=0.002; fixed-effects model: RR: 1.62, 95% CI: 1.19–2.20, *P*=0.002; *I^2^* = 4%). Adjustment of estimated glomerular filtration rate (RR: 1.73, *P*=0.02) or B-type natriuretic peptide (BNP) or N-terminal pro-BNP (RR: 1.83, *P*=0.02) did not significantly affect the result. A trend of stronger association between higher baseline circulating galectin-3 and increased risk of all-cause mortality after TAVR was observed in studies with an enzyme-linked fluorescent assay (ELFA) (RR: 3.04, *P*=0.003) compared with those with an enzyme-linked immunosorbent assay (ELISA) (RR: 1.42, *P*=0.04; *P* for subgroup difference =0.06).

**Conclusion:** Higher circulating galectin-3 before the procedure may predict all-cause mortality of AS patients after TAVR.

## Introduction

Aortic stenosis (AS) is an important valvular heart disease characterized by progressive narrowing of aortic heart valve [1]. With the aging of global population, AS caused by aging-related degeneration of the aortic heart valve is expected to increase, which may finally lead to heart failure (HF) in these patients [1,2]. Medicines targeting HF has limited efficacy to improve the symptoms related to AS, and surgical aortic valve replacement (SAVR) may be ultimate treatment to relieve the symptoms related to AS in these patients [3]. However, high-risk patients with severe AS may not be able to tolerate conventional SAVR, such as the elderly patients [4]. For these patients, transcatheter aortic valve replacement (TAVR) has emerged as an alternative treatment option which confers equal therapeutic efficacy of SAVR [5,6]. Moreover, TAVR is not restricted to inoperable and high-risk patients, but also an alternative to surgery in intermediate-risk and low-risk patients [5]. However, some patients after TAVR remain to have poor survival, so early identification of patients who may not benefit from TAVR is of clinical importance, preferably from clinical parameters before the procedure [7,8].

Increasing evidence showed that circulating galectin-3, an emerging biomarker of myocardial fibrosis, immune activation, and enhanced inflammation, has been involved in the pathogenesis of many cardiovascular diseases [9,10]. Previous studies have shown that increased circulating galectin-3 is associated with the severity of cardiac dysfunction and ventricular remodeling in patients with myocardial infarction [11], acute or chronic HF [12], or even in general population without overt cardiovascular disease [13]. In addition, higher galectin-3 has also been proposed as a prognostic factor for patients with HF [12]. Moreover, previous studies suggested an important role of galectin-3 in the pathogenesis and progression of AS. In an experimental study of rat model of pressure overload, vascular galectin-3 overexpression was detected, which was accompanied by enhanced aortic valve fibrosis, inflammation, as well as greater expression of calcification markers [14]. The other study in rat model of AS induced by supravalvular aortic banding also showed enhanced cardiac galectin-3 expression, which paralleled higher myocardial fibrosis and inflammation [15]. Another study confirmed the overexpression of galectin-3 in aortic valves from AS patients, and suggested the mechanistic role of galectin-3 in AS, such as stimulating deposition of extracellular matrix, facilitating inflammation, and modulating osteogenic differentiation of valvular interstitial cells [16]. More importantly, blockage of galectin-3 has been proposed as a new therapeutic approach to delay the progression of AV calcification in AS [16]. These findings indicated that galectin-3 overexpression may play key role in the pathogenesis and progression of AS [16]. Interestingly, early studies have proposed that increased circulating galectin-3 before TAVR may predict poor survival in AS patients [17]. However, only one study showed a significant result [17], while the remaining did not support a significant association between circulating galectin-3 and all-cause mortality after TAVR [18–21]. The sample sizes of these studies are relatively small, which may not be statistically adequate to reach a significant finding. Therefore, in the present study, we aimed to systematically evaluate the association between preprocedural galectin and mortality in AS patients receiving TAVR by pooling the data of previous studies in a meta-analysis.

## Methods

The Meta-analysis of Observational Studies in Epidemiology (MOOSE) [22] and Cochrane’s Handbook [23] guidelines were followed during the designing, performing, and reporting of the meta-analysis. This article does not contain any studies with human participants performed by any of the authors.

### Literature search

Systematic search of electronic databases of PubMed, Cochrane’s Library, and Embase were performed to identify potentially relevant studies from the index date to 20 November 2019. The combined terms were entered into the databases as a single search, as (‘galectin-3’ OR ‘galectin 3’) AND (‘Aortic stenosis’ OR ‘Transcatheter Aortic Valve Implantation’ OR ‘Transcatheter Aortic Valve Replacement’ OR ‘TAVI’ OR ‘TAVR’). We used these keywords search strategy instead of those searched as ‘text words’ or as ‘Mesh terms’ to retrieve more comprehensive records. The full search strategy for PubMed was provided in *Supplementary File S1*. We used this extensive search strategy to avoid missing of potentially relevant studies. The search was limited to human studies, and no language restriction was applied. Besides, we also studied the reference lists of related original studies and review articles using a manual approach.

### Study selection

The inclusion criteria were: (1) full-length articles reporting longitudinal follow-up studies, including cohort studies, post-hoc analyses of randomized controlled trials and nested case–control studies; (2) enrolled adult patients with AS that scheduled for TAVR; (3) measured circulating galectin-3 before TAVR; (4) patients were grouped according to baseline level of galectin-3; (5) investigated the association between baseline galectin-3 and all-cause mortality risk after TAVR during a minimal follow-up duration of 3 months; and (4) reported the relative risk for this association after adjustment of potential confounding factors. Review articles, preclinical studies, and studies irrelevant to the purpose of current meta-analysis were excluded.

### Data extraction and quality evaluation

Two authors independently performed database search, data extraction, and study quality assessment according to predefined criteria. If discrepancies occurred, they were solved by consensus between the two authors or discussion with the corresponding author. The following data were extracted: (1) study information: name of first author, publication year, and study country; (2) study design characteristics; (3) patient characteristics: sample size, age, sex, prevalence of diabetes, and proportions of patients with coronary artery disease (CAD) at baseline; (4) echocardiographic parameters reflecting the AS severity of study participants, including aortic valve area and mean transaortic gradient; (5) access route of TAVR; (6) measuring methods and cut-off for galectin-3; (7) follow-up duration and number of mortality cases during follow-up; and (8) adjusted confounding factors. The Newcastle–Ottawa Scale was used as an instrument for study quality evaluation [24]. This scale ranges from 1 to 9 stars, and assesses study quality mainly regarding three domains, including study group selection, between-group comparability, and validation of the outcome of interest.

### Statistical analyses

A risk ratio (RR) with corresponding 95% confidence interval (CI) was used as the main measure for the association between preprocedural circulating galectin-3 and mortality risk after TAVR. Data of RRs and their corresponding standard errors (SEs) were calculated from 95% CIs or *P*-values, and a logarithmical transformation was performed to stabilize variance and normalize the distribution [23]. The Cochrane’s Q-test was performed to evaluate the heterogeneity, and the *I^2^* statistic was also estimated [25]. An *I^2^* > 50% indicates significant heterogeneity. Both the fixed- and the random-effects models were used for the meta-analysis [23]. By omitting one individual study at a time, we performed sensitivity analyses to test the robustness of the results [26]. Moreover, whether adjustment of estimated glomerular filtrating rate (eGFR) or B-type natriuretic peptide (BNP) or N-terminal pro-BNP (NT-proBNP), a known prognostic marker for mortality after TAVR [27], that may change the result was also evaluated in subgroup analyses. Moreover, results of studies with different measuring methods for galectin-3 were also compared. The potential publication bias was initially detected by visual inspection of the symmetry of funnel plots, then complemented with the Egger’s regression asymmetry test [28]. RevMan (Version 5.1; Cochrane Collaboration, Oxford, U.K.) software was used for the meta-analysis.

## Results

### Literature search

**Figure 1:**
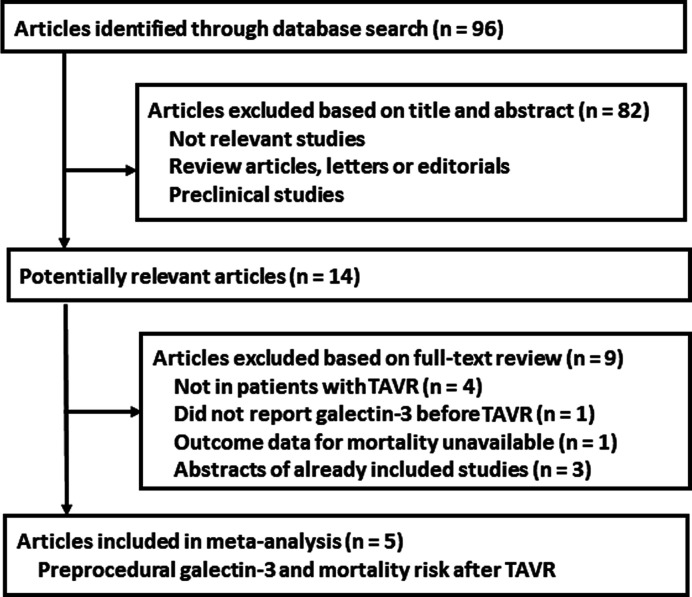
Flowchart of database search and study identification

Figure 1 shows the literature search process. Briefly, 96 articles were obtained via initial search of the PubMed, Cochrane’s Library, and Embase databases, and 82 were excluded through screening of the titles and abstracts mainly because they were not relevant to the purpose of the meta-analysis. Subsequently, 14 records underwent full-text review. Of these, nine were further excluded because four of them did not include patients with TAVR, one did not report baseline galectin-3 value before TAVR, one did not provide mortality data, and the remaining three were abstracts of already included studies. Finally, we included five studies in this meta-analysis [17–21].

### Study characteristics and quality evaluation

The characteristics of the studies were presented in Table 1. All of them were prospective cohort studies, and performed in Germany [17,21], the United States [18,20], and Poland [19], respectively. These studies were published between 2014 and 2019. Overall, 854 patients with AS that received TAVR were included. The mean age of the patients varied from 78 to 84 years, with percentage of males ranging from 45 to 59%. Diabetic patients accounted for 26–46% of the included patients, and 55–86% of the included patients were with CAD. The mean aortic valve areas varied from 0.62 to 0.74 cm^2^, and the mean transaortic gradient ranged between 44 and 50 mmHg. Circulating galectin-3 was measured with enzyme-linked fluorescent assay (ELFA) [17,19] or enzyme-linked immunosorbent assay (ELISA) [18,20,21] before TAVR. In two studies [17,19], a galectin-3 threshold value of 17.8 ng/ml was applied, based on the U.S. Food and Drug Administration—cleared assay labeling of galectin for risk stratification in patients with HF [29]. In another two studies, median [18,20] for galecitin-3 were applied for grouping. The remaining one study used cut-off value of galectin-3 based on the maximally selected rank statistics [21]. During a mean follow-up duration of 1–1.9 years, 131 deaths occurred (15.3%). Potential confounding factors including age, sex, diabetic status, previous CAD, comorbidities, eGFR, and BNP or NT-proBNP were adjusted to a variable degree among the included studies. The Newcastle–Ottawa Scale scores of the included studies ranged from 6 to 9, indicating generally good study quality (Table 2).

**Table 1:** Characteristics of the included studies

Study	Country	Design	Sample size	Mean age	Male	DM	CAD	AV area	Mean transaortic gradient	TF access for TAVR	Gal-3 measurement	Gal-3 cutoff	Follow-up duration	Deaths	Variables adjusted	NOS
				years	%	%	%	cm^2^	mmHg	%		ng/ml	years			
Baldenhofer, 2014	Germany	PC	101	78	45	46	61	0.74	44	100	ELFA	17.8 (U.S. FDA-CHF)	1	16 (16)	Age, gender, eGFR, and NT-proBNP	7
Lindman, 2015	U.S.A.	PC	183	NA	NA	NA	86	NA	NA	42	ELISA	18.8 (median)	1.9	54 (29)	Age, gender, eGFR, DM, COPD, NYHA class, and aortic gradient	8
Kim, 2017	U.S.A.	PC	112	84	59	32	55	0.62	50	82	ELISA	18.4 (median)	1	20 (18)	Age, gender, BMI, STS scores, and BNP	7
Bobrowska, 2017	Poland	PC	19	NA	NA	NA	NA	NA	NA	NA	ELFA	17.8 (U.S. FDA-CHF)	1.4	4 (21)	Age, gender, and eGFR	6
Rheude, 2019	Germany	PC	439	81	55	26	74	NA	44	100	ELISA	8.7 (the maximally selected rank statistics)	1	37(8)	Age, gender, EuroSCORE, comorbidities, eGFR, NT-proBNP, and aortic gradient	8

Abbreviations: AV, aortic valve; BMI, body mass index; COPD, chronic obstructive pulmonary disease; DM, diabetes mellitus; Gal-3, galectin-3; NOS, the Newcastle–Ottawa Scale; NYHA, New York Heart Association; PC, prospective cohort study; ROC, receiver operating characteristic curve; STS, Society of Thoracic Surgeons; TF, transfemoral; U.S. FDA-CHF, the U.S. Food and Drug Administration—cleared assay labeling of galectin-3 for risk stratification in patients with heart failure.

**Table 2:** Details of study quality evaluation by the Newcastle–Ottawa Scale

Study	Representativeness of the exposed cohort	Selection of the non- exposed cohort	Ascertainment of exposure	Outcome not present at baseline	Control for age and gender	Control for other confounding factors	Assessment of outcome	Enough long follow-up duration	Adequacy of follow-up of cohorts	Total
Baldenhofer, 2014	**0**	**1**	**1**	**1**	**1**	**0**	**1**	**1**	**1**	**7**
Lindman, 2015	**1**	**1**	**1**	**1**	**1**	**1**	**1**	**1**	**0**	**8**
Kim, 2017	**0**	**1**	**1**	**1**	**1**	**1**	**0**	**1**	**1**	**7**
Bobrowska, 2017	**0**	**1**	**1**	**1**	**1**	**0**	**1**	**1**	**0**	**6**
Rheude, 2019	**1**	**1**	**1**	**1**	**1**	**1**	**1**	**1**	**0**	**8**

### Results of meta-analysis

**Figure 2:**
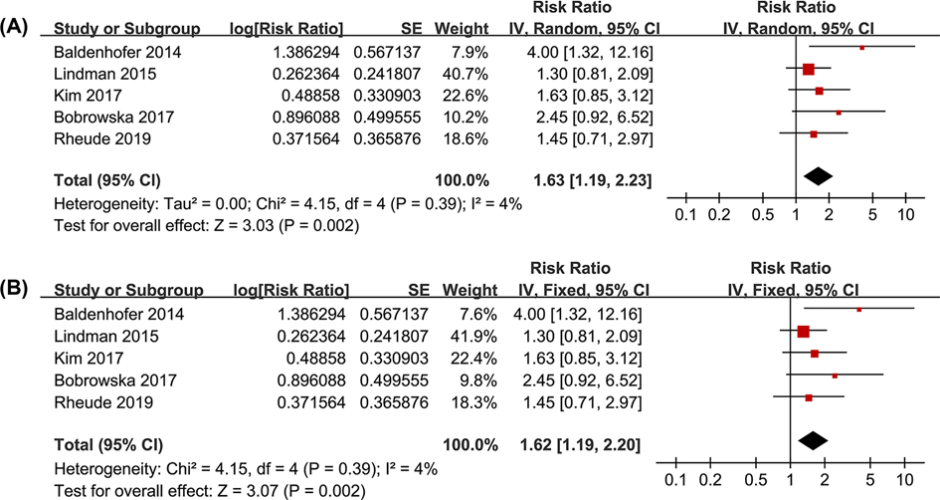
Forest plots for the meta-analysis of the association between circulating galectin-3 at baseline and all-cause mortality risk after TAVR (**A**) Pooled results using a random-effects model; (**B**) Pooled results using a fixed-effects model.

**Figure 3:**
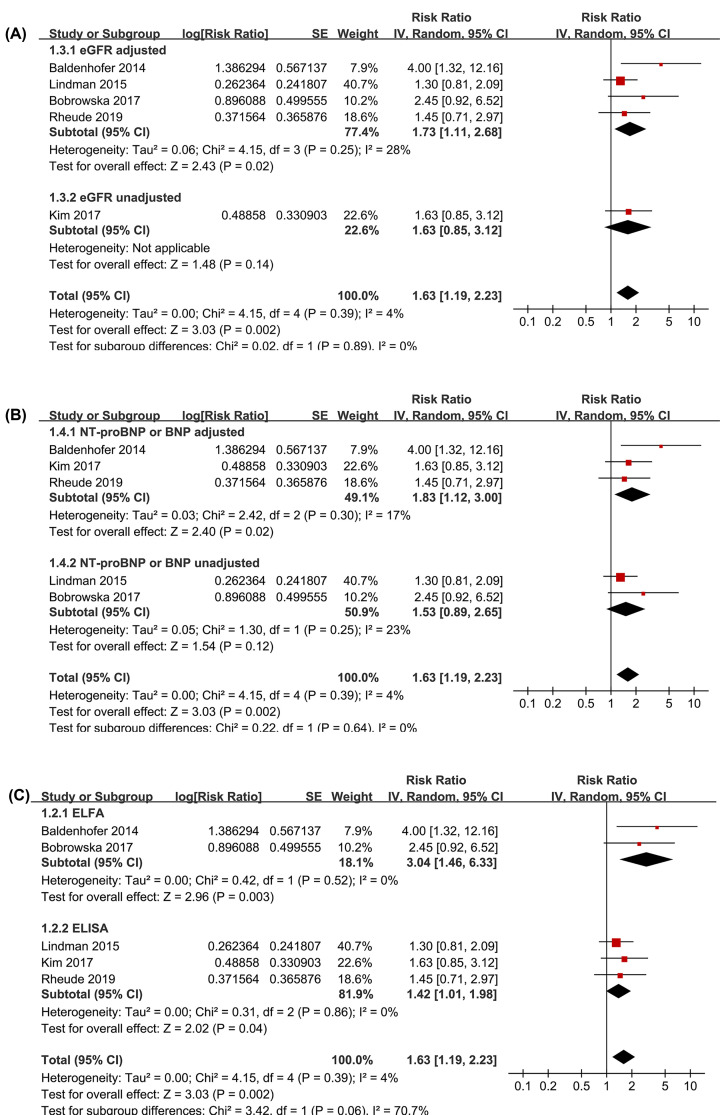
Subgroup analyses for the meta-analysis of the association between circulating galectin-3 at baseline and all-cause mortality risk after TAVR (**A**) Subgroup analyses according to the adjustment of eGFR. (**B**) Subgroup analyses according to the adjustment of BNP or NT-proBNP. (**C**) Subgroup analyses according to the gelectin-3 measuring methods of ELFA or ELISA.

Pooled results of all included studies using a random-effects model and fixed-effects model; both showed that patients with higher baseline circulating galectin-3 were associated with an increased risk of all-cause mortality after TAVR (random-effects model: RR: 1.63; 95%; CI: 1.19–2.23; *P*=0.002; Figure 2A and fixed-effects model: RR, 1.62; 95% CI: 1.19–2.20; *P*=0.002; Figure 2B) with no significant heterogeneity (*P* for Cochrane’s Q test = 0.39, *I^2^* = 4%). Sensitivity analyses by omitting one study at a time did not significantly change the results (RR: 1.50–1.89, *P* all <0.05; Table 3). Subgroup analyses showed that the association between higher galectin-3 and increased all-cause mortality risk after TAVR remained significant in studies with adjustment of eGFR (RR: 1.73, 95% CI: 1.11–2.68, *P*=0.02; *I^2^* = 28%; Figure 3A) and in studies with adjustment of BNP or NT-proBNP (RR: 1.83, 95% CI: 1.12–3.00, *P*=0.02; *I^2^* = 17%; Figure 3B). A trend of stronger association between higher baseline circulating galectin-3 and increased risk of all-cause mortality after TAVR was observed in studies with ELFA (RR: 3.04, *P*=0.003) compared with those with ELISA (RR: 1.42, *P*=0.04; *P* for subgroup difference =0.06; Figure 3C).

**Table 3:** Sensitivity analysis

Study excluded	Meta-result: RR (95% CI)	*P* for overall results	*I^2^*	*P* for Cochrane’s Q test
Baldenhofer, 2014	1.50 [1.09, 2.07]	0.01	0	0.71
Lindman, 2015	1.89 [1.27, 2.83]	0.002	0	0.43
Kim, 2017	1.73 [1.11, 2.68]	0.02	28%	0.25
Bobrowska, 2017	1.57 [1.11, 2.23]	0.01	11%	0.34
Rheude, 2019	1.76 [1.16, 2.68]	0.008	26%	0.26

### Publication bias

**Figure 4:**
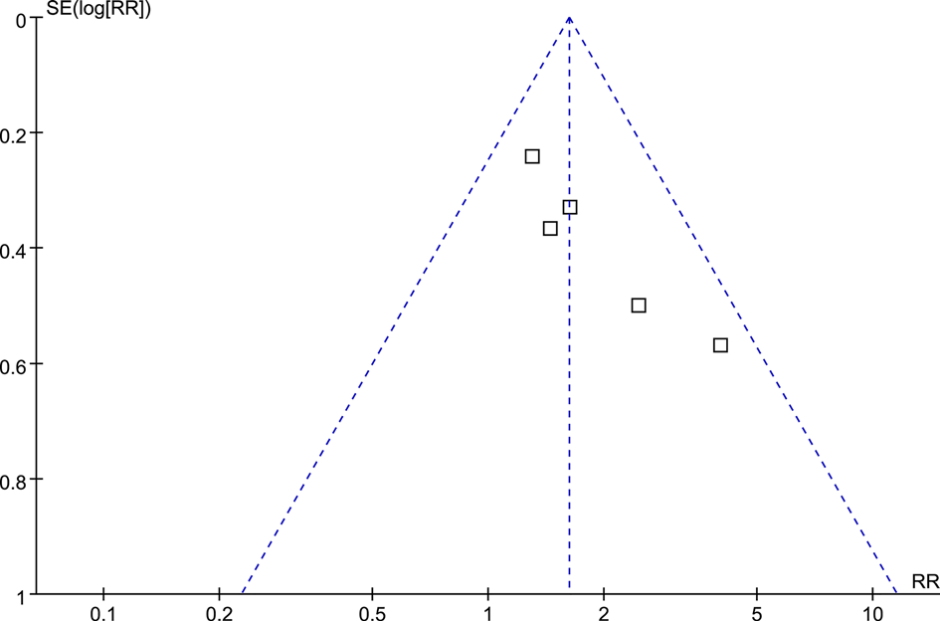
Funnel plots for the publication bias underlying the meta-analysis of the association between circulating galectin-3 at baseline and all-cause mortality risk after TAVR

The funnel plots for the meta-analysis of the association between baseline circulating galectin-3 and risk of all-cause mortality after TAVR were shown in Figure 4. These plots were symmetrical on visual inspection, suggesting low risk of publication bias. Egger’s regression test was not performed since only five studies were available.

## Discussion

By summarizing the current evidence from observational studies, our meta-analysis showed higher circulating galectin-3 before the procedure may predict all-cause mortality of AS patients after TAVR. Specifically, the association between galectin-3 and mortality after TAVR remained significant in studies adjusted for eGFR and in studies adjusted for BNP or NT-proBNP, while a trend of stronger association was found in studies with ELFA compared with those with ELISA. Taken together, these results suggested that circulating galectin-3 at baseline may be a predictor of all-cause mortality after TAVR.

To the best of our knowledge, our study seems to be the first meta-analysis focusing on the association between circulating galectin-3 and mortality in AS patients after TAVR. The strengths of our study included the following. First, we only included studied with multivariate adjustment, which therefore may suggest an independent association between preprocedural circulating galectin-3 and mortality after TAVR. Moreover, we used sensitivity analysis to confirm the robustness of the findings, which was not primarily driven by either of the included study. Finally, subgroup analyses were performed to evaluate the influence of study characteristics on the results, which showed that association between higher galectin-3 and increased all-cause mortality after TAVR was consistent even after adjustment of eGFR, BNP, or NT-proBNP. These results support that circulating galectin-3 before the procedure may predict the all-cause mortality for AS patients undergoing TAVR.

It has been observed that circulating galectin-3 is increased in patients with renal dysfunction [30]. Moreover, impaired renal function has been related to poor prognosis after TAVR [31,32]. Therefore, it could be inferred that renal dysfunction may confound the association between increased circulating galectin-3 and poor survival after TAVR. However, our subgroup analyses showed that the association between increased circulating galectin-3 and higher all-cause mortality in AS patients after TAVR remained significant in studies with adjustment of eGFR, indicating that the association between galectin-3 and prognosis after TAVR is unlikely to be mediated by kidney dysfunction. Similarly, previous studies showed that BNP or NT-proBNP is an important prognostic biomarker for poor survival after TAVR [27]. Our subgroup analyses indicated that the association between increased circulating galectin-3 and higher all-cause mortality in patients after TAVR remained significant in studies with adjustment of BNP or NT-proBNP. These findings suggest that measuring of galectin-3 may confer additional prognostic significance on the basis of BNP or NT-proBNP for AS patients after TAVR. Moreover, a trend of stronger association between increased circulating galectin-3 and higher all-cause mortality in patients after TAVR was found in studies with ELFA compared with those with ELISA, suggesting that measuring methods for galectin-3 may affect the study outcome. Studies are needed to determine the optimal measuring methods and cut-off values regarding patients after TAVR. Currently, it remains unknown whether galectin-3 is a simple biomarker or an important factor involved in the progression of AS after TAVR. Experimental studies remain rare regarding the role of galectin-3 in pathogenesis and progression of AS. However, the downstream pathophysiological events of galectin-3, including myocardial fibrosis, inflammation, and oxidative stress, have all been implicated in the development of AS [33]. Interestingly, a previous study showed that galectin-3 is overexpressed in aortic valves in AS patients that underwent SAVR [16]. Moreover, in cultured valvular interstitial cells, galecin-3 induced expression of inflammatory, fibrotic, and osteogenic markers through the extracellular signal-regulated kinase 1 and 2 pathway, leading to calcification in AS [16].

Our study has limitations. First, only five cohort studies were available for the meta-analysis, and the sample size of the included studies was also limited. Accordingly, results of subgroup analyses should be interpreted with caution. Large-scale prospective cohort studies are needed to validate these findings. Second, the cut-off values for circulating galectin-3 and follow-up durations varied among the included studies. The optimal measuring methods and cut-off value for circulating galectin-3 at baseline to predict mortality risk after TAVR remains to be determined, and no studies have been performed to compare the association between galectin-3 measured by ELFA and ELISA directly. Future studies are warranted. Third, only galectin-3 before procedure was investigated in our study. It has been proposed that reduction in circulating galectin-3 after TAVR may predict better survival [34]. The influence of dynamic changes of galectin-3 during the periprocedural period of TAVR on mortality risk in these patients deserves further investigation. Fourth, as a meta-analysis of observational studies, a causative association between higher galectin-3 and poor survival in patients after TAVR could not be derived based on our findings. Future studies are needed to determine whether galectin-3 was involved in the pathogenesis and progression of AS or it is simply a biomarker. In addition, although multivariable adjusted data were combined, we could not exclude the residual factors that may confound the association between galectin-3 and mortality after TAVR, such as the comorbidities of the patients, as well as the administration of statins [35]. Besides, although studies based on multivariate adjusted analysis are effective to control potential confounding factors, application this inclusion criterion may lead to the exclusion of univariate studies with negative findings, which may introduce selection bias to the meta-analysis. More importantly, previous large registries of TAVR patients have shown that comorbidities are the most important predictors of mortality after TAVR [36,37]. In this regard, an individual participant data (IPD)-level meta-analysis is recommended. However, due to the lack of access to the IPD data of these studies, such a study was unable to be performed by us at the current stage. Finally, whether galectin-3 predicts long-term mortality risk after TAVR should be evaluated in future studies.

## Conclusion

In conclusion, results of meta-analysis suggested that higher circulating galectin-3 before the procedure may predict all-cause mortality of AS patients after TAVR. These results should be validated in large-scale prospective studies and future studies are needed to elucidate the role of galectin-3 in the progression of AS after TAVR.

## Abbreviations

AS: aortic stenosis
BNP: B-type natriuretic peptide
CAD: coronary artery disease
CI: confidence interval
eGFR: estimated glomerular filtrating rate
ELFA: enzyme-linked fluorescent assay
ELISA: enzyme-linked immunosorbent assay
HF: heart failure
IPD: individual participant data
NT-proBNP: N-terminal pro-BNP
RR: risk ratio
SAVR: surgical aortic valve replacement
TAVR: transcatheter aortic valve replacement
